# Clinical and structural insights into the rare but oncogenic *HER2*-activating missense mutations in non-small cell lung cancer: a retrospective ATLAS cohort study

**DOI:** 10.1007/s12672-024-01154-2

**Published:** 2024-07-16

**Authors:** Guangjian Yang, Runze Liu, Pei Li, Yaning Yang, Yajie Wang, Huiqing Mao, Xiaoyong Tang

**Affiliations:** 1 Department of Respiratory Medical Oncology, Shandong First Medical University and Shandong Academy of Medical Sciences, Shandong Cancer Hospital and Institute, No.440 Jiyan Road, Jinan, 250117 Shandong China; 2 Department of Radiation Oncology, Shandong First Medical University and Shandong Academy of Medical Sciences, Shandong Cancer Hospital and Institute, Jinan, 250117 Shandong China; 3 Department of Medical Oncology, National Cancer Center/National Clinical Research Center for Cancer/Cancer Hospital, Chinese Academy of Medical Sciences and Peking Union Medical College, Beijing, 100021 China

**Keywords:** *HER2*, Missense mutation, Non-small cell lung cancer, Tyrosine kinase inhibitor, Chemotherapy, Targeted therapy

## Abstract

**Background:**

Unlike human epidermal growth factor receptor 2 (*HER2*) amplification or exon 20 insertions, missense mutations in the extracellular domain (ECD), transmembrane domain (TMD), and intracellular domain (ICD) of the *HER2* protein have been implicated as oncogenic in non-small cell lung cancer (NSCLC). However, their molecular subtypes, structural disparities, and clinical responses to current medical treatments, particularly *HER2*-targeted tyrosine kinase inhibitors (TKIs), remain unclear in NSCLC and warrant investigation.

**Methods:**

A real-world observational ATLAS study was conducted to gather and analyze therapeutic outcomes of chemotherapy or TKIs for heterogeneous *HER2* missense mutations in NSCLC. Computational models of typical ECD, TMD, and ICD mutations were utilized to explore their structural variances.

**Results:**

We screened 37 eligible patients with *HER2*-activating missense mutations, of which 35 patients who had received chemotherapy or *HER2*-targeted TKIs as first-line therapy were available for response assessment. The median progression-free survival (PFS) for chemotherapy was 4.43 months (95% confidence interval [CI], 3.77–5.10), with an objective response rate (ORR) of 26.1% (6/23) and a disease control rate (DCR) of 17/23 (73.9%). The administration of afatinib, dacomitinib, and pyrotinib, *HER2*-targeted TKIs, achieved a median PFS of 4.65 months, with an ORR of 33.3% (4/12) and a DCR of 83.3% (10/12). Molecular modeling and computational simulations of ECD, TMD, and ICD mutations revealed their distinct structural characteristics.

**Conclusion:**

In comparison to chemotherapy, *HER2*-targeted TKIs demonstrated similar activity and PFS benefits for *HER2*-activating missense mutations in NSCLC.

## Introduction

The human epidermal growth factor receptor 2 (*HER2*, *ERBB2*) has been identified as an oncogenic driver in non-small cell lung cancer (NSCLC), with approximately 3% of patients with NSCLC harboring heterogeneous *HER2* aberrations [1, 2]. As a receptor tyrosine kinase of the *ErbB* family, *HER2* lacks a known ligand, and is instead activated by heterodimerization with other *ErbB* family members such as epidermal growth factor receptor (*EGFR*, *HER1*), *HER3*, and *HER4* [1, 3]. Among its three functional forms, *HER2* protein overexpression and gene amplification have been reported in 6–35% and 10–20% of NSCLC cases, respectively, whereas *HER2* genomic alterations are documented to occur in only 2–4% [2, 4]. Exon 20 insertion has been reported to be the most common *HER2-*activating alteration in NSCLC, which tends to affect the ATP-binding pocket of the *HER2* receptor, leading to steric hindrance against conventional *HER2*-targeted tyrosine kinase inhibitors (TKIs) [1, 5]. Additionally, several missense mutations in the extracellular domain (ECD, encoding amino acids 23–652), the transmembrane domain (TMD, encoding amino acids 653–675), and the intracellular domain (ICD, encoding amino acids 676–1255) of the *HER2* protein have been identified as functional and oncogenic in NSCLC. These mutations can promote tumor proliferation and progression and may be targetable by TKIs, as supported by in vitro and in vivo evidence [6–9].

Various ECD or TMD missense mutations have been associated with sensitivity to *HER2*-targeted inhibitors in NSCLC [7–9]; however, it is not yet a validated hallmark to predict the response to *HER2*-targeted agents. An increasing number of *HER2*-activating missense mutations are being identified and described by multiplex next-generation sequencing (NGS) in NSCLC; however, current evidence regarding *HER2*-activating missense mutations in NSCLC consists mainly of case reports or studies with small sample sizes focusing on specific mutations due to their rare frequency.

Furthermore, there is a lack of research reporting comprehensive *HER2* missense alteration subtypes and their clinical responses to *HER2*-targeted TKIs in NSCLC, with a focus on highlighting their molecular features to provide clinical reference as a benchmark. Therefore, our real-world ATLAS cohort study was conducted to evaluate the preliminary activity of *HER2*-targeted TKIs for heterogeneous *HER2* missense alterations in metastatic NSCLC, aiming to reveal their discrepant structures.

## Materials and methods

### Patients and data collection

The medical records and clinical data of patients with metastatic NSCLC and *HER2*-activating missense mutations were retrospectively reviewed at the National Cancer Center/Cancer Hospital, Chinese Academy of Medical Sciences (Beijing), and Shandong Cancer Hospital and Institute (Jinan, Shandong Province) between September 2017 and April 2023. *HER2*-activating missense mutations were detected via NGS testing utilizing the Illumina sequencing system in institutional laboratories or qualified third-party genetic testing companies certified by the national quality system. Formalin-fixed, paraffin-embedded tissue samples or plasma were used. The last follow-up occurred on April 10, 2023. The requirement for informed consent was waived as this was a retrospective observational study.

### Response assessment

Tumor response was evaluated as complete response (CR), partial response (PR), stable disease (SD), or progressive disease (PD) by analyzing computed tomography images of the chest and abdomen, and brain magnetic resonance imaging according to the Response Evaluation Criteria in Solid Tumors version 1.1. Progression-free survival (PFS) was considered the time from treatment initiation to the first evidence of disease progression or death from any cause. The objective response rate (ORR) was determined as the number of patients with confirmed CR and PR, whereas the disease control rate (DCR) was calculated as the proportion of confirmed CR, PR, and SD.

### Computational structure modeling

The homology modeling procedure was conducted using the Molecular Operating Environment software (MOE, version 2020.01) “Homology Model” program, starting with a multiple sequence alignment of the primary structures. The three-dimensional (3D) structures of p.S310F and p.S310Y were generated through comparative modeling, using the *HER2* ECD deposited in the RCSB Protein Data Bank [PDB, code: 3WLW] as a template. Similarly, the 3D structure of p.V659E was modeled using the TMD of *EGFR* and *HER2* receptors deposited in the RCSB Protein Data Bank [PDB, code: 2KS1] as a template, whereas the structures of p.L755P and p.V842I were built based on the 2.25 Å resolution crystal structure of the kinase domain of human *HER2* deposited in the RCSB Protein Data Bank [PDB, code: 3PP0].

### Statistical analyses

Statistical analyses were performed using SPSS software, version 20.0 (IBM Corp., Armonk, NY, USA). Continuous variables were summarized using medians and ranges, and categorical variables were described by frequency and percentage. The Kaplan–Meier method was used to analyze PFS, and 95% confidence intervals (CIs) were estimated using the Cox proportional regression model. A *p*-value of < 0.05 was considered statistically significant. Survival curves were plotted using GraphPad Prism version 5.0 (GraphPad Software Inc., San Diego, CA, USA).

## Results

### Patient characteristics and molecular subtypes

A total of 37 eligible patients with heterogeneous *HER2*-activating missense mutations were screened, of which 35 had received chemotherapy or *HER2*-targeted TKIs as first-line therapy and were available for response assessment. The clinicopathological characteristics of patients in this study was summarized in Table 1. All patients had lung adenocarcinomas, with 48.6% (n = 18) being females and 54.1% (n = 20) never-smokers. The majority underwent NGS testing on tumor tissue (n = 33, 89.2%). ECD mutations were identified in 13 patients (35.1%), including p.V308M (n = 1), p.S310F (n = 7), p.S310Y (n = 2), p.S335C (n = 1) in exon 8, and p.R456C (n = 2) in exon 12. TMD mutations were noted in five patients (13.5%), including p.V659E (n = 4) and V664F (n = 1) in exon 17. The remaining 19 patients (51.4%) harbored ICD mutations, including p.G727A in exon 18 (n = 1), p.L755P (n = 10), p.L755S (n = 2), and p.D769Y (n = 1) in exon 19, p.G776V (n = 1), p.R811Q (n = 1) in exon 20, and V842I (n = 1), p.T862A (n = 1), p.L841I plus L869R (n = 1) in exon 21.

**Table 1:** Clinicopathological characteristics of NSCLC patients with *HER2* missense mutations

Characteristics	N (%)
Average age (years)	58
Gender	
Male	19 (51)
Female	18 (49)
Pathology	
Adenocarcinoma	37 (100)
Smoking history	
Never	20 (54)
Current/former	17 (46)
Brain metastases at baseline	
Absence	32 (86)
Presence	5 (14)
NGS specimen	
Tumor tissue	33 (89)
Plasma	4 (11)
HER2 missense mutation	
ECD	13 (35)
TMD	5 (14)
ICD	19 (51)
First-line treatment	
Chemotherapy	23 (62)
*HER2*-targeted inhibitor	12 (32)
Not available	2 (6)

*ECD* extracellular domain, *TMD* transmembrane domain, *ICD* intracellular domain

### Responses of first-line therapy for *HER2* missense mutations

**Fig. 1:**
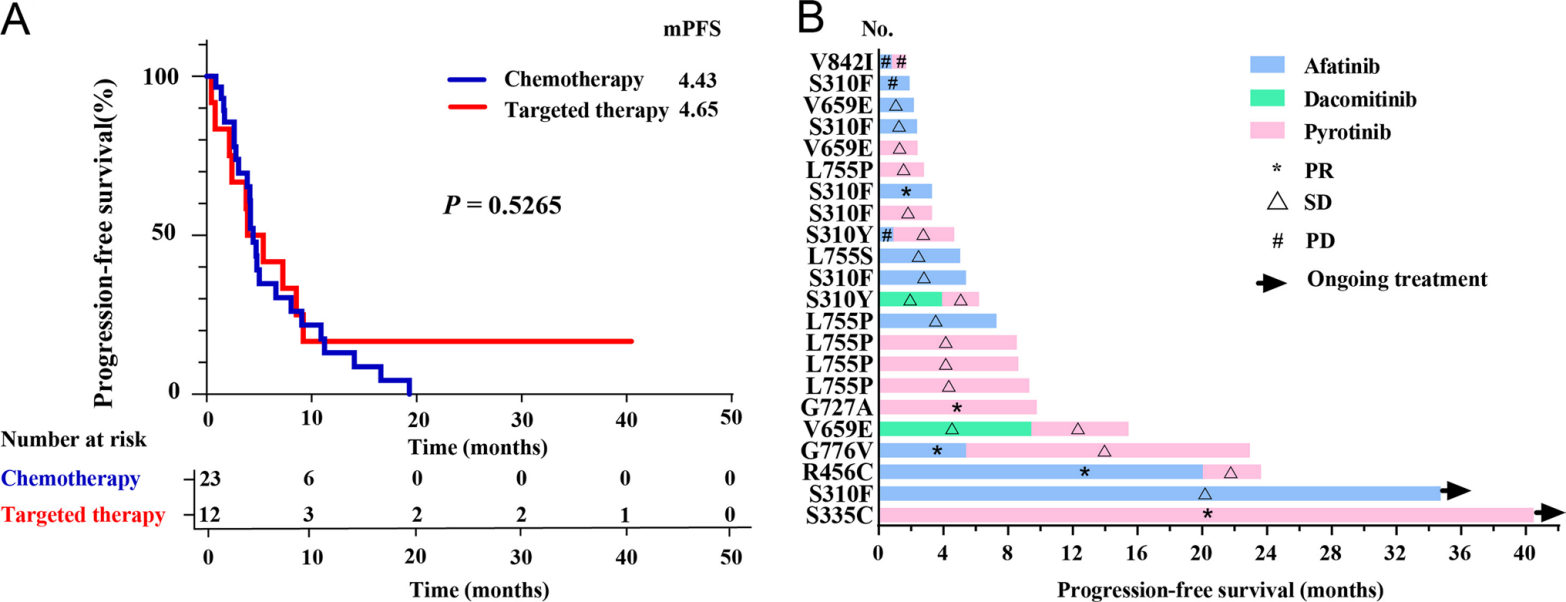
Kaplan–Meier curves of PFS in patients treated with chemotherapy or *HER2*-targeted TKIs in the first-line setting (**A**). Swimmer plot of PFS benefit from *HER2*-targeted TKIs (**B**)

Among the 35 patients, 23 (62.2%) received first-line platinum-based chemotherapy, including chemotherapy alone (n = 5), chemotherapy in combination with anti-vascular endothelial growth factor (*VEGF*) antibody bevacizumab (n = 10), chemotherapy in combination with programmed cell death protein 1 (PD-1) inhibitor (n = 6), or chemotherapy in combination with bevacizumab and PD-1 inhibitor (n = 2). The median PFS for chemotherapy-based options was 4.43 (95% CI 3.77–5.10) months, with an ORR of 26.1% (6/23) and a DCR of 73.9% (17/23). Detailed chemotherapy regimen, genetic mutation and response for each case was summarized in Table 2. *HER2*-targeted TKIs (afatinib, dacomitinib, pyrotinib) were administered to another 12 patients (32.4%) as first-line therapy. They achieved a median PFS of 4.65 months (*P* = 0.527, Fig. 1A), with an ORR of 33.3% (4/12) and a DCR of 83.3% (10/12) compared with chemotherapy-based option. Table 3 summarized the targeted outcomes of heterogeneous *HER2* missense mutations, and a swimmer plot depicting the PFS benefit of *HER2*-targeted TKIs is shown in Fig. 1B.

**Table 2:** Response to first-line chemotherapy-based option in patients with NSCLC harboring *HER2* missense mutations

No	Age/sex	Exon/domain	Protein alteration	Nucleotide change	Regimen	Best response	PFS (months)
1	59/F	19/ICD	p.L755S	c.2264 T > C	PEM + CBP	SD	3.0
2	74/M	19/ICD	p.L755S	c.2264 T > C	PEM + LBP	SD	2.6
3	68/F	19/ICD	p.L755P	c.2263_2264delinsCC	PEM + DDP + BEV	SD	3.8
4	73/F	19/ICD	p.L755P	c.2263_2264delinsCC	PEM + CBP + BEV	SD	4.1
5	61/M	19/ICD	p.L755P	c.2263_2264delinsCC	PEM + CBP + BEV + Camrelizumab	PR	4.4
6	56/F	19/ICD	p.L755P	c.2263_2264delinsCC	PEM + CBP + Sintilimab	PR	4.2
7	57/M	19/ICD	p.L755P	c.2263_2264delinsCC	PEM + CBP + Pembrolizumab	PR	2.8
8	46/M	19/ICD	p.L755P	c.2263_2264delinsCC	PEM + CBP + BEV	SD	5.0
9	63/F	19/ICD	p.L755P	c.2263_2264delinsCC	PEM + CBP + Camrelizumab	SD	4.7
10	64/F	19/ICD	p.L755P	c.2263_2264delinsCC	PEM + CBP + BEV + Camrelizumab	PR	9.1
11	60/M	19/ICD	p.D769Y	c.2305G > T	PEM + CBP + BEV	PD	1.4
12	61/M	18/ICD	p.G727A	c.2180G > C	Nab-*paclitaxel* + CBP	SD	3.8
13	34/F	17/TMD	p.V659E	c.1976_1977delinsAA	PEM + CBP + BEV	SD	4.1
14	54/F	17/TMD	p.V659E	c.1976_1977delinsAA	PEM + DDP + BEV	SD	10.9
15	65/F	21/ICD	p.T862A	c.2584A > G	PEM + CBP + Camrelizumab	SD	4.8
16	68/M	21/ICD	p.V842I	c.2524G > A	PEM + CBP + BEV	SD	16.6
17	54/M	21/ICD	p.L841I p.L869R	c.2521C > A c.2606 T > G	PEM + CBP + Toripalimab	PD	1.7
18	65/M	12/ECD	p.R456C	c.1366C > T	PEM + CBP	SD	14.1
19	57/F	12/ECD	p.R456C	c.1366C > T	PEM + DDP + BEV	SD	6.6
20	61/M	8/ECD	p.S310F	c.929C > T	PEM + CBP + Sintilimab	PR	19.3
21	67/F	8/ECD	p.S310F	c.929C > T	PEM + CBP + BEV	SD	11.2
22	68/M	8/ECD	p.S310F	c.929C > T	PEM + CBP	SD	1.6
23	56/M	8/ECD	p.S310F	c.929C > T	PEM + CBP + BEV	PR	8.0

*ECD* extracellular domain, *F* female, *M* male, *ICD* intracellular domain, *PD* progressive disease, *PFS* progression-free survival, *PR* partial response, *SD* stable disease, *TMD* transmembrane domain, *PEM* pemetrexed, *CBP* carboplatin, *LBP* lobaplatin, *DDP* cisplatin, *BEV* bevacizumab

**Table 3:** Response to *HER2*-targeted TKIs in patients with NSCLC harboring heterogeneous *HER2* missense mutations

No.	Age/Sex	Exon/domain	Protein alteration	Nucleotide Change	TKI	Best response	PFS (months)	Treatment line
1	50/M	8/ECD	p.S335C	c.1003A > T	Pyrotinib^a^	PR	40.5	1
2	36/M	8/ECD	p.S310F	c.929C > T	Afatinib	PD	1.9	3
3	67/F	8/ECD	p.S310F	c.929C > T	Afatinib^a^	SD	34.7	1
4	61/M	8/ECD	p.S310F	c.929C > T	Pyrotinib	SD	3.3	2
5	67/F	8/ECD	p.S310F	c.929C > T	Afatinib	SD	3.3	2
6	68/M	8/ECD	p.S310F	c.929C > T	Afatinib	SD	5.4	2
7	71/M	8/ECD	p.S310F	c.929C > T	Afatinib	SD	2.4	2
8	37/M	8/ECD	p.S310Y	c.929C > A	Dacomitinib	SD	3.9	1
					Pyrotinib	SD	2.3	4
9	68/M	8/ECD	p.S310Y	c.929C > A	Pyrotinib	SD	3.8	1
					Afatinib	PD	0.9	2
10	57/F	12/ECD	p.R456C	c.1366 C > T	Afatinib	SD	20.0	2
					Pyrotinib	SD	3.6	4
11	34/F	17/TMD	p.V659E	c.1976_1977delinsAA	Dacomitinib	SD	9.4	2
					Pyrotinib	SD	6.0	3
12	62/M	17/TMD	p.V659E	c.1976_1977delinsAA	Afatinib	SD	2.2	1
13	71/F	17/TMD	p.V659E	c.1976_1977delinsAA	Pyrotinib	SD	2.4	1
14	61/M	18/ICD	p.G727A	c.2180G > C	Pyrotinib	PR	9.8	2
15	46/M	19/ICD	p.L755P	c.2263_2264delinsCC	Pyrotinib	SD	2.8	3
16	49/F	19/ICD	p.L755P	c.2263_2264delinsCC	Afatinib	SD	7.3	1
17	56/F	19/ICD	p.L755P	c.2263_2264delinsCC	Pyrotinib	SD	6.9	3
18	59/F	19/ICD	p.L755S	c.2264 T > C	Afatinib	SD	5.0	2
19	68/F	19/ICD	p.L755P	c.2263_2264delinsCC	Pyrotinib	SD	8.6	2
20	52/F	19/ICD	p.L755P	c.2263_2264delinsCC	Pyrotinib	PR	8.5	1
21	31/F	20/ICD	p.G776V	c.2327G > T	Afatinib	PR	5.4	1
					Pyrotinib	SD	17.5	2
22	68/M	21/ICD	p.V842I	c.2524G > A	Pyrotinib	PD	0.9	2
					Afatinib	PD	0.8	3

*ECD* extracellular domain, *F* female, *M* male, *ICD* intracellular domain, *PD* progressive disease, *PFS* progression-free survival, *PR* partial response, *SD* stable disease, *TKI* tyrosine kinase inhibitor, *TMD* transmembrane domain

^a^Ongoing treatment

### Efficacy of *HER2*-targeted TKIs for *HER2* missense mutations

**Fig. 2:**
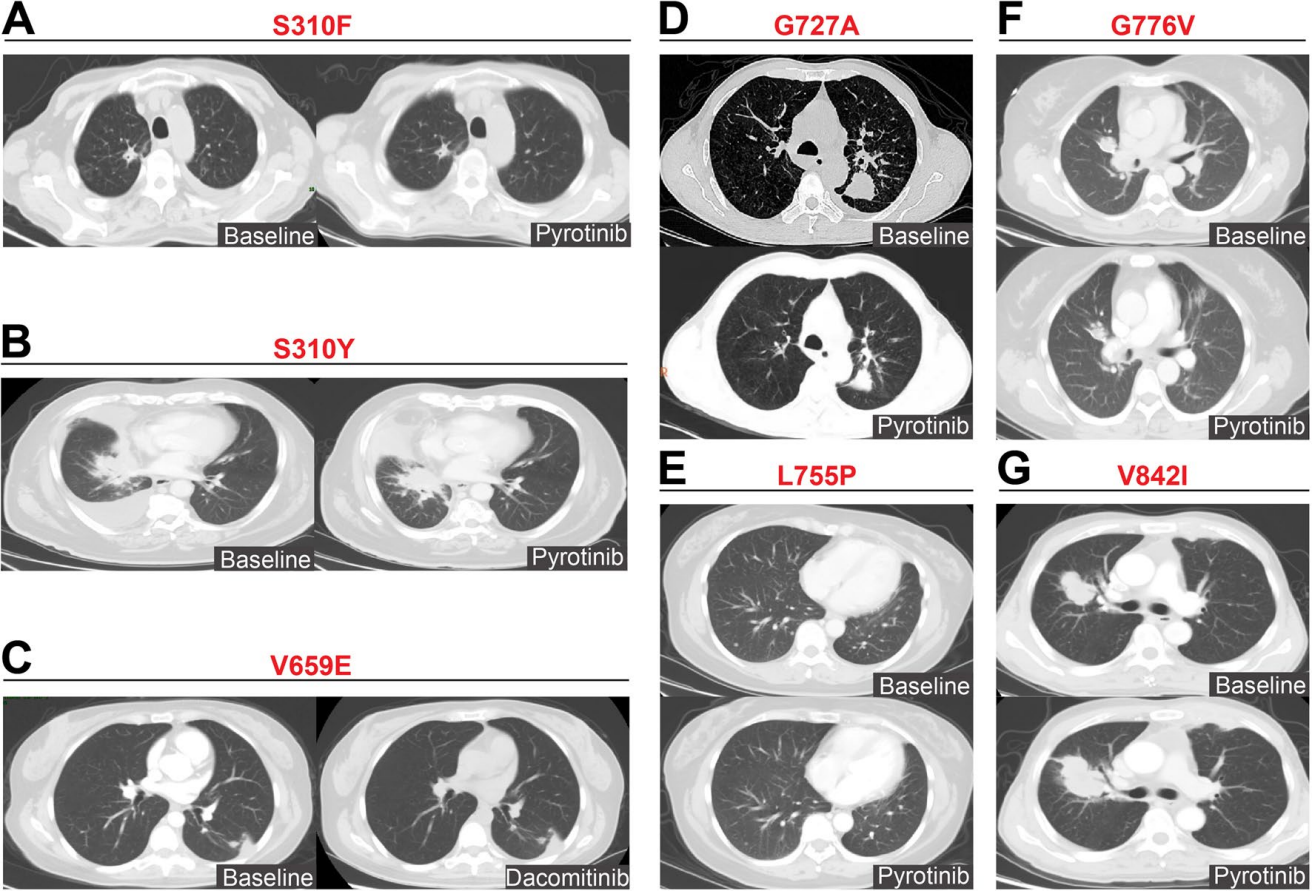
Case series revealing targeted responses to *HER2*-TKIs of variable *HER2* missense alterations. S310F (**A**) and S310Y (**B**) in exon 8 of ECD, V659E in exon 17 of TMD (**C**), and kinase domain alterations in the ICD, including G727A in exon 18 (**D**), L755P in exon 19 (**E**), G776V in exon 20 (**F**), and V842I in exon 21 (**G**)

Among the six patients with p.S310F mutation, one patient exhibited intrinsic PD (PFS of 1.9 months) to afatinib, whereas another exhibited SD, with an ongoing PFS of 34.7 months to afatinib in the first-line setting. The remaining four patients responded with SD to afatinib or pyrotinib, with PFS ranging between 2.4 and 5.4 months (Fig. 2A). One patient with the p.S310Y mutation exhibited SD (PFS of 3.9 months) to dacomitinib and SD (PFS of 2.3 months) to pyrotinib. Similarly, another patient with the p.S310Y mutation exhibited SD (PFS of 3.8 months) to first-line pyrotinib but experienced rapid PD (PFS of 0.9 months) to afatinib (Fig. 2B). For the three patients with the p.V659E alteration, two received pyrotinib (PFS of 2.4 months) and afatinib (PFS of 2.2 months), both achieving SD as the best response. Dacomitinib (PFS of 9.4 months) and pyrotinib (PFS of 6.0 months, Fig. 2C) were administered to the third patient, also with SD as the best response.

For the nine patients with ICD missense alterations, diverse alteration subtypes in the kinase domain spanning exon 18–21 were observed, with variable responses to *HER2*-targeted TKIs. A patient with the exon 18 p.G727A mutation exhibited PR to pyrotinib, with a PFS of 9.8 months (Fig. 2D). Another patient harboring the exon 19 p.L755P mutation exhibited PR and PFS of 8.5 months to pyrotinib (Fig. 2E). A patient with exon 20 p.G776V mutation achieved significant PFS benefit of 17.5 months to pyrotinib (Fig. 2F). However, a patient harboring exon 21 p.V842I mutation experienced intrinsic PD to both pyrotinib (PFS of 0.9 month, Fig. 2G) and afatinib (PFS of 0.8 month).

### Structural analysis

**Fig. 3:**
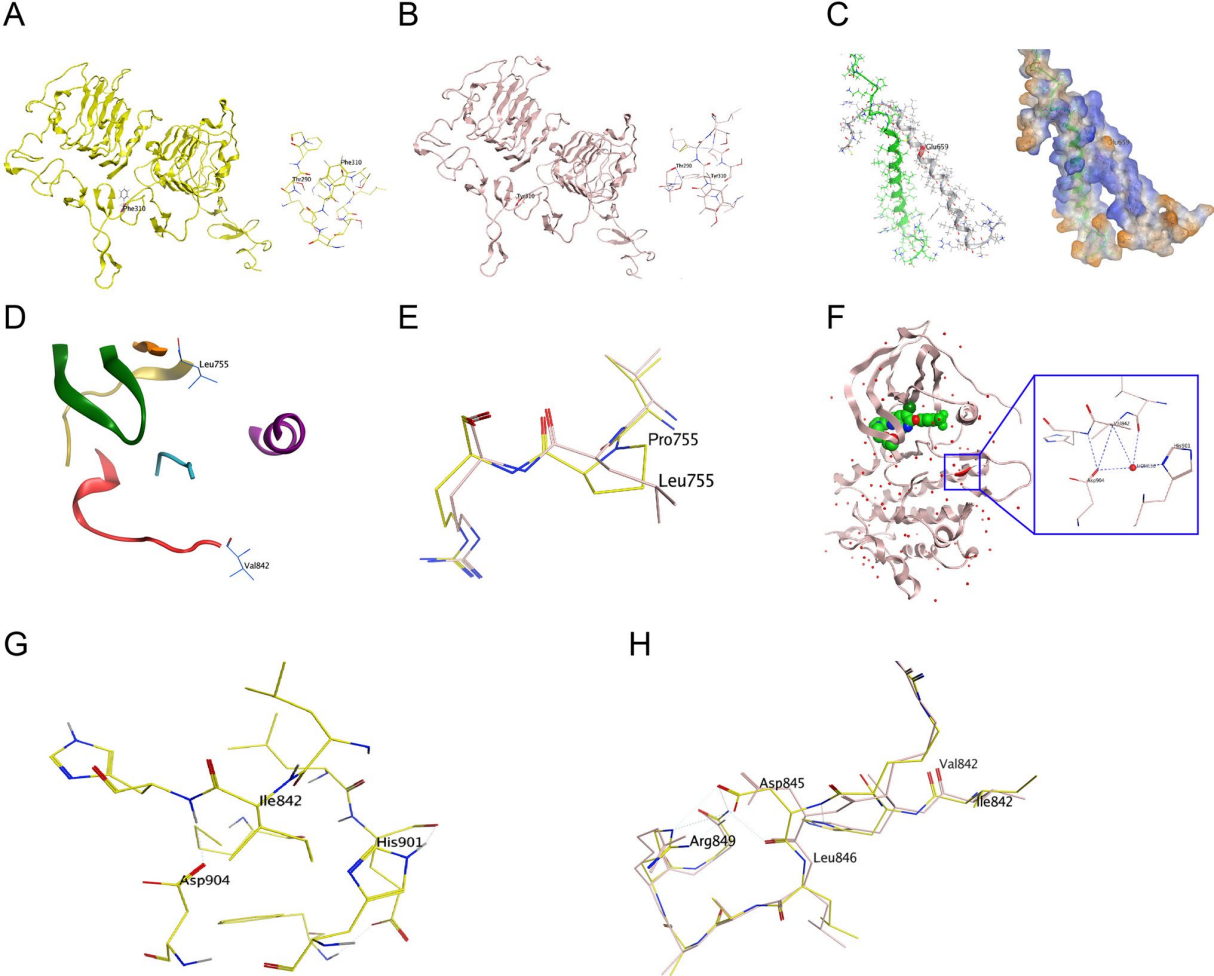
Crystal structures and molecular interactions between S310F (**A**) and S310Y (**B**). Homology models of V659E (**C**) in the TMD of *EGFR*/*HER2* dimer. Residue Leu755 and Val842 in the kinase domain of *HER2* (**D**). Homology modelling of Leu755 and Pro755 (**E**). Val842 close to the catalytic subunit interacts with Asp904 and His901 via H-bonds (**F**), and there was no interaction between Ile842 and Asp904 or His901 (**G**). Modification in amino acid positions of the catalytic unit induced of V842I mutation (**H**)

In the crystal structure of the wild-type *HER2* receptor, the amino acid residue Ser310 is situated in the ECD, exhibiting an H-bond interaction with Thr290. However, in the p.S310F conformation (Fig. 3A), Phe310 lacks interaction with Thr290. Similarly, in the p.S310Y conformation (Fig. 3B), Tyr310 also does not interact with Thr290. The homology structures of the hydrophilic residue Glu659 in the TMD of the *EGFR*/*HER2* dimer are depicted in Fig. 3C. Residue Leu755 in the kinase domain of *HER2* is proximal to the Hyd1 region (marked in orange, Fig. 3D), an important hydrophobic region encoding amino acids Ala751-Ile752-Lys753. Residues within Hyd1 engage in hydrophobic contact with the adenine ring of ATP, forming a hydrophobic pocket. Homology modeling revealed that both leucine (Leu755) and proline (Pro755) are hydrophobic amino acids, exhibiting little difference in 3D structural conformation (Fig. 3E). However, Val842 is in close proximity to the catalytic subunit (marked in red, Fig. 3D), encoding amino acids His843 to Leu852, and Val842 interacts with Asp904 and His901 via several H-bonds (Fig. 3F), contributing to the stability of the *HER2* protein. No interactions between the mutated Ile842 and Asp904 and His901 were observed (Fig. 3G). The conformation of V842I is less stable than that of wild-type Val842 because of the absence of molecular interaction, potentially resulting in structural changes in the *HER2* protein. Moreover, the V842I alteration eliminates the interaction between Val842 itself and other protein residues, contributing to modifications in amino acid positions of the catalytic unit, such as Asp845, Leu846, and Arg849 (Fig. 3H).

## Discussion

**Fig. 4:**
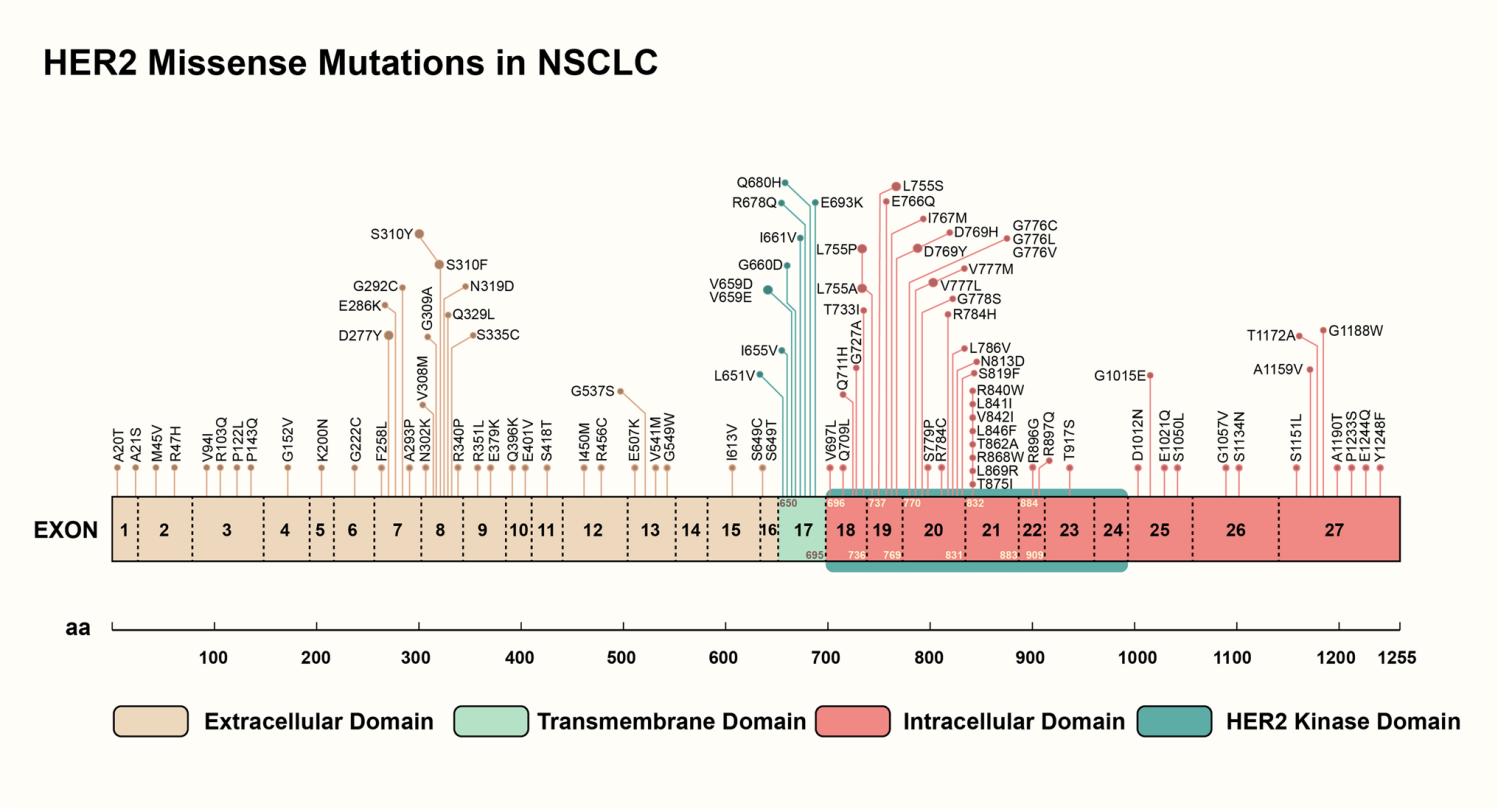
Missense mutation subtypes spreading in each exon of *HER2* receptor in NSCLC documented and reported in the Cosmic, cBioPortal and Foundation Medicine sequencing datasets

This real-world ATLAS study offers valuable insights into the molecular signature and clinical activity of chemotherapy or *HER2*-targeted TKIs against uncommon *HER2*-activating missense mutations in NSCLC. Currently, we present the most comprehensive atlas for *HER2*-activating missense mutations in NSCLC (Fig. 4), encompassing molecular subtypes of various missense mutations across each exon of the *HER2* receptor in NSCLC, documented or reported in datasets such as Cosmic, cBioPortal, and Foundation Medicine sequencing [9]. Certain types of *HER2* missense alterations in NSCLC have been identified as oncogenic and have shown sensitivity to *HER2*-targeted inhibitors [9–17]. Our observations demonstrate that *HER2*-targeted TKIs, including afatinib, dacomitinib, and pyrotinib, exhibit similar PFS (median, 4.65 *vs.* 4.43 months) and ORRs (33.3% *vs.* 26.1%) compared to chemotherapy for these alterations as first-line therapy, respectively.

*HER2* somatic alterations have been reported across various human cancer types and are linked with activating functions that drive oncogenesis akin to gene amplification and *HER2* protein overexpression, which may similarly confer drug sensitivity to *HER2*-targeted TKIs [18–20]. In NSCLC, exon 20 insertions in the *HER2* kinase domain have been extensively studied regarding their molecular features and responses to clinical treatment [1, 2, 5]. However, other heterogeneous missense mutations in NSCLC in different regions of *HER2*, such as ECD, TMD, and ICD, have received less attention and investigation regarding their potential for activating *HER2* and their responses to currently available *HER2*-directed TKIs. The most common ECD mutation, p.S310F, is a well-known activating alteration promoting *HER2* noncovalent dimerization and mediating *HER2* hyperphosphorylation [8, 21]. Few reports on p.S310F have mentioned favorable responses to trastuzumab and lapatinib [22, 23]. Evidence has also validated that p.S310F induces structural changes in the *HER2* extracellular subdomain II in vitro and forms an active heterodimer with *EGFR*, thereby abolishing reactivity to pertuzumab [2]. Another ECD mutation, p.S310Y, has been identified in patients with NSCLC [8, 24], with reported sensitivity to afatinib in lung adenocarcinoma [12]. Functional analysis suggests that *HER2* ECD mutants p.S310F and p.S310Y can be activated by mechanisms involving C-terminal phosphorylation elevation and formation of disulfide-linked dimers, leading to hydrophobic interactions between the newly introduced 310F or 310Y and neighboring molecules, thus promoting noncovalent dimerization and *HER2* kinase activation, which may benefit from *HER2*-targeted therapy [8].

In our ECD mutation cohort, five patients harboring p.S310F exhibited SD to afatinib or pyrotinib, with one patient exhibiting an ongoing PFS of 34.7 months with first-line afatinib therapy. Additionally, two patients harboring the p.S310Y mutation exhibited SD to dacomitinib or pyrotinib. These results suggest that pan-*ErbB* TKIs exhibit promising activity for *HER2* ECD mutations in NSCLC. To the best of our knowledge, this is the first report on dacomitinib and pyrotinib activities for p.S310F and p.S310Y mutations. Our structural analysis provides meaningful evidence regarding p.S310F and p.S310Y mutations and offers supplementary insights into the molecular hydrophobicity and interactions of *HER2* ECD missense mutations. Additionally, we report a valuable case with the p.R456C mutation in exon 12, another rare ECD missense alteration which has not been previously reported, achieving a favorable PFS of 20 months on afatinib.

Furthermore, the *ErbB* family TMD is crucial for receptor activation, affecting downstream signaling activity independently of kinase domain mutations [25]. Typical *HER2* TMD mutations, such as p.V659E, are well-known NSCLC oncogenic drivers [13, 26, 27]. The largest research cohort of more than 8000 lung adenocarcinoma samples to date reported the detection of 0.15% (15/8551) of *HER2* TMD mutations at amino acid residues V659 or G660 in NSCLC, including p.V659E, p.V659D, p.G660D, and p.G660R, along with other non-V659/G660 TMD mutations, including p.V664F, p.V665M, and p.I675M [7]. Ou et al. performed structural analysis, revealing that V659/G660 TMD mutations stabilized *HER2* homodimerization and heterodimerization to maintain *HER2* in the active conformation, with treatment using afatinib resulting in durable clinical responses in three of four patients [7]. A Chinese multi-center cohort study also revealed the comprehensive profiles and real-world evidence of *HER2* TMD mutation treatment in NSCLC, with a total prevalence of 0.18% (14/7812) and 0.14% (11/7812) for the p.V659E alteration. This study indicated that TMD mutations were associated with more advanced stages (*p* < 0.001) and poorer overall survival (median, 10.0 *vs.* 61.6 months, hazard ratio = 7.9, *p* < 0.001) than non-TMD mutations [28]. Favorable PFS outcomes with targeted therapy, including afatinib (up to 16 months), and better responses to pyrotinib were noted among cohort patients, suggesting that pyrotinib effectively inhibits the p.V659E mutation. Structural analysis of binding affinity subsequently demonstrated increased binding ability to pyrotinib and afatinib toward the p.V659E mutation [28]. In our study, which included three patients harboring p.V659E, our data correlated well with the abovementioned evidence, further implying that, in addition to afatinib and pyrotinib, dacomitinib is a promising TKI candidate for the p.V659E mutation. We observed that the p.V659E mutation altered the hydrophobicity of residue Val659, which may explain the discrepant binding affinity to the abovementioned *HER2*-targeted TKIs.

Among *HER2* ICD alterations in NSCLC, the in-frame insertion in exon 20 (A775_G776insYVMA) is the most frequent subtype [1, 2, 6]. Several studies have revealed that exon 20 insertions exhibit relatively poor responses to traditional pan-*ErbB* TKIs, including afatinib, dacomitinib, and neratinib, with low ORRs of 3.8–11.5% and PFS ranging from 3 to 5.5 months [16, 29, 30]. The ZENITH20-2 study reported a median PFS of 5.5 months and an improved ORR of 35.1% in patients with NSCLC harboring *HER2* exon 20 insertions treated with the novel pan-*ErbB* TKI poziotinib [31]. A significantly improved PFS (median, 8.2 months) and ORR of 55% was reported in patients with NSCLC and *HER2* mutations treated with trastuzumab deruxtecan (T-DXd, DS-8201) in the DESTINY-Lung01 study [32]. Prospective studies have increasingly focused on *HER2* exon 20 insertions in advanced NSCLC; however, little is known regarding the targeted outcomes of pan-*ErbB* TKIs for the uncommon *HER2* ICD missense alterations in real-world settings. Our PEARL study revealed a median PFS of 5.8 months, an ORR of 23.0%, and a DCR of 85.1% with pyrotinib among *HER2*-mutated patients with NSCLC. Those with *HER2* missense mutations exhibited a notable PFS benefit (median, 12.2 *vs.* 6.8 *vs.* 5.2 months) compared with *HER2* amplification and exon 20 non-YVMA insertions, respectively [33]. Based on a single-arm, phase II study on *HER2*-mutated advanced NSCLC, pyrotinib facilitated a median PFS of 6.9 months and an ORR of 30% as second-line or above therapy. Several patients with *HER2* kinase domain missense mutations, including p.G776R, p.G776C, p.V777L, p.L755P, were enrolled, with an ORR of 25.0% in four patients with the p.L755P mutation [34].

In NSCLC, exon 18 p.G727A, exon 20 p.G776V, and exon 21 p.V842I mutations in the *HER2* receptor kinase domain have been recorded in the Foundation Medicine or cBioPortal dataset and recognized as oncogenic [9]; however, their responses to *HER2* inhibitors remain unclear. Moreover, while the p.L775P and p.L755S missense mutations have been considered oncogenic and sensitive to neratinib, poziotinib, and pyrotinib [15, 16, 34], their responses to afatinib and antitumor capability confirmed by pyrotinib require further study. Our ATLAS cohort study provided initial evidence of activity with *HER2*-targeted TKIs for these *HER2* rare missense mutations. We observed that both the p.G727A and p.G776V mutations exhibited favorable responses to pyrotinib, with significant PFS benefits of 9.8 and 17.5 months, respectively. However, the p.V842I mutation exhibited de novo drug resistance to both pyrotinib and afatinib. This resistance might be attributed to its location near the catalytic subunit and the lack of molecular interaction between Ile842 and other residues, resulting in modifications in amino acid positions of the catalytic unit and structural changes in the *HER2* protein, as indicated by in silico structural analysis. In our cohort, patients with the p.L755P and p.L755S mutations treated with afatinib or pyrotinib exhibited favorable responses and PFS benefits, similar to what has been previously reported [34]. Furthermore, it was found that both residue Leu755 and Pro755 were hydrophobic amino acids, with little difference in their activity to TKI binding and 3D structural conformation.

*HER2*-mutant lung cancers have a clinical course with a high incidence of brain metastases [35]. One of the reason is the absence of effective, targeted therapies for *HER2* mutations, and currently the first-line standard recommendation for the treatment of lung cancer with *HER2* mutations still remains cytotoxic chemotherapy. In addition, *HER2-*mutant cancers are associated with increased expression of the chemokine receptor C-X-C chemokine receptor type 4 (CXCR4). CXCR4 and its ligand, stromal-derived-factor-1 (SDF-1; also called CXCL12), may drive metastatic trafficking to the brain [36, 37]. The frequency of brain metastases at diagnosis was similar in NSCLC patients carrying *HER2* mutations (19%), compared to patients with *KRAS* (24%) and *EGFR* mutations (31%) [35]. However, lung cancer patients with *HER2* mutations developed more brain metastases during treatment than patients with *KRAS* (28% *vs.* 8%, hazard ratio [HR] 5.2, *P* < 0.001) and *EGFR* mutations (28% *vs* 16%, HR 1.7, *P* = 0.06) [35]. Yang et al. also demonstrated that *HER2* exon 20 YVMA insertion is associated with a higher incidence of lifetime brain metastasis, with estimated 12-month brain metastasis incidence as 40.2% compared with 3.6% in the non-YVMA group in patients with advanced NSCLC and *HER2* kinase domain mutations [38]. In our cohort study, among the total 37 patients harboring *HER2*-activating missense mutations, only five (14%) presented baseline brain metastasis. After the failure of first-line therapy, eight patients occurred brain metastasis, with a brain metastasis frequency of 35%, which is in accordance with observations discussed above.

T-DXd is currently the sole approved *HER2*-targeted therapy for previously treated NSCLC patients with *HER2* mutations. The randomized, blinded multicenter phase II trial DESTINY-Lung02 showed considerable and enduring antitumor responses of T-DXd in *HER2*-mutated NSCLC patients, with ORR of 49% and 56% at doses of 5.4 and 6.4 mg/kg, regardless of *HER2* mutation type, amplification status, and prior treatment [39]. In several countries, T-DXd, rather than *HER2*-targeted TKIs, has become the standard choice for second-line treatment in NSCLC patients with *HER2* mutations. Unfortunately, none of the patients with *HER2*-activating missense mutations involved in this study were treated with T-DXd. The accessibility of T-DXd in the mailand of China, financial considerations from patients and their family members, and the current cognition of HER2-activating missense mutations both from lung cancer patients and doctors are all factors that might explain for this status.

Sugimoto et al. had reported plasma cell-free DNA (cfDNA) sequencing in patients with NSCLC showed relatively high sensitivity for detecting gene mutations but low sensitivity for gene fusions and *MET* exon 14 skipping, with a positive percent agreement of plasma cfDNA sequencing compared with tissue DNA and RNA assays were 77% (*EGFR*, 78%; *KRAS*, 75%; *BRAF*, 85%; *HER2*, 72%) and 47% (*ALK*, 46%; *RET*, 57%; *ROS1*, 18%; *MET*, 66%), respectively [40]. Plasma cfDNA sequencing could be useful for detecting oncogenic alterations only when tissue assay is unavailable, and it could not fully replace tissue assays for oncogenic alterations detection, especially when the quality and quantity of tissue samples are acceptable for genomic analysis. In this cohort study, four patients performed liquid biopsy using circulating cfDNA owing to the inadequate tumor tissue or the will of non-invasive testing. They were detected to carry p.L755P, p.V659E, p.S310Y, and p.L841I with p.L869R missense mutations, respectively. To some degree, liquid biopsy for *HER2*-mutant lung cancer using circulating cfDNA is a reasonable alternative.

Despite the ATLAS study investigated the clinical activity of currently available *HER2*-targeted TKIs for heterogeneous *HER2* missense mutations in NSCLC, along with valuable evidence from in silico structural analysis, several limitations must be noted. Firstly, this was a retrospective real-world study prone to selection bias. Additionally, the small sample size of patients with *HER2* missense mutations prevented us from further investigating molecular variants and their precise activity toward *HER2*-targeted TKIs. The structural differences and molecular interactions were explained with regard to specific missense subtypes; however, this was an exploratory analysis based on computational structure and dynamics simulation and may not fully represent all possible reasons. Further clinical evidence, including cell lines and patient-derived xenograft models, is warranted to corroborate our findings and draw clear conclusions.

In conclusion, compared to conventional chemotherapy, currently available *HER2*-targeted TKIs exhibited similar efficacy and reasonable activity for patients with NSCLC harboring *HER2*-activating missense mutations. Awareness of these extremely rare but oncogenic *HER2* missense alterations may advance promising targeted therapy in NSCLC for this entity.

## Data Availability

The data that support the findings of this study are not openly available due to reasons of sensitivity and are available from the corresponding author upon reasonable request.
